# Supplementary material for: Genome analyses reveal population structure and a purple stigma color gene candidate in finger millet
Source: Nat Commun. 2023 Jun 21;14:3694. doi: 10.1038/s41467-023-38915-6 (PMC10284860; doi:10.1038/s41467-023-38915-6)
Supplement: Supplementary file 1 — Supplementary Information [file 41467_2023_38915_MOESM1_ESM.docx]

**Genome analyses reveal population structure and a purple stigma color gene candidate in finger millet**

Devos *et al.*

## **Supplementary Note 1. Assessment of genome quality**

During the initial round of Illumina polishing (~65x of Illumina reads, 2x150, 400 bp insert), there were a total of 419 homozygous single nucleotide polymorphisms (SNPs) and 18,845 homozygous insertion/deletions (INDELs) for a total of 19,264 errors (1 error every 57,635 bp). A closer examination of the Illumina polishing revealed that only 1.041 Gb out of a total of 1.11 Gb sequence fell within the depth ranges for calling errors, leaving 68,378,311 bases not accessible to polishing calling. If we assume the same error rate for the callable bases, we likely missed out on fixing a total of 1,186 errors. Aligning the Illumina reads to the polished genome revealed that a total of 139 homozygous SNPs and 136 homozygous INDELs persisted. This enables us to compute an overall genome quality score (QV). If we include the 1,186 errors in the uncalled bases, which also likely persisted, then we have a total of 1,461 errors remaining in the polished genome. That results in an error rate of 1 error every 759,995 bp, which translates to a genome quality score (QV score) of 58.8.

## **Supplementary Note 2. Characteristics of the A and B genome chromosomes**

Chromosome numbering was based on the nomenclature used in the genetic maps^1^. The B-genome chromosomes are between 16% and 37% larger than the homoeologous A-genome chromosomes (Supplementary Fig. 2). Alignments of the KNE 796-S v1.0 release chromosomes against the nine A genome and nine B genome linkage groups in the newly generated genetic maps (Supplementary Data 13) showed that the majority of finger millet chromosomes are metacentric or submetacentric, except chromosomes 4A and 4B which are acrocentric (Supplementary Fig. 3). Accession KNE 796-S carries two terminal reciprocal translocations, between homoeologous chromosomes 6A and 6B, and between 9A and 9B, as evidenced by the alignment of *Eleusine indica* (AA genome) reads to regions ~46,980,000 – end on chromosome 6B and 0 – ~3,140,000 on chromosome 9B (Supplementary Fig. 1). The homoeologous translocations on chromosomes 6 and 9 encompass 21.3% of 6A, 18.7% of 6B, 6.8% of 9A and 5.6% of 9B. The 6A/6B translocation, the 9A/9B translocation and an interstitial reciprocal 2A/2B translocation had previously been identified in the MD-20 x Okhale-1 linkage map^1^ with the 6A/6B translocation characterizing both parents, while the 2A/2B and 9A/9B translocations differentiated the two parents. The 9A/9B translocation also differentiated the wild and cultivated parents of a second mapping population^2^. Examination of the deletion breakpoints in non-balanced 9A and 9B chromosomes generated through adjacent meiotic disjunction in F_1_ hybrids that are heterozygous for the translocation^1,2^ narrowed down the translocation breakpoints to the region 3,110,118 – 3,175,631 bp on 9A and 3,473,943 – 3,773,483 bp on 9B. Further manual inspection of the *E. indica* read alignments to the KNE 796-S v1.0 reference genome, and the SNP distribution in those regions across the resequenced finger millet germplasm and in the *E. indica* acc. HZ-2018 genome assembly^3^ delineated the breakpoint to a 46 bp segment that was highly conserved between chromosomes 9A and 9B. Mining of the finger millet resequencing reads for SNPs that flanked the breakpoints and/or PCR amplification across the breakpoints on 9A and 9B showed that the translocation was absent in wild accessions, had mixed presence in Ethiopian germplasm and was, with the exception of one cultivar, uniformly present in the cultivated accessions from countries other than Ethiopia (Supplementary Data 3). The data strongly suggest that the 9A/9B translocation occurred during or immediately after finger millet domestication.

##

## **Supplementary Note 3. Repeat annotation and analysis**

The total repeat content of the KNE 796-S v1.0 finger millet genome assembly amounts to 61.3%. The absolute and relative amounts of different types of repeat elements in the assembly are provided in the main text in Table 1. Long terminal repeat retrotransposons (LTR-RTs), in particular Gypsy elements, represent the largest repeat component. Because the B genome is some 24% (116 Mb) larger than the A genome (Supplementary Fig. 2), we checked whether a differential amount of LTR-RTs in the A and B genomes could explain the size difference. To account for the homoeologous translocations, the region 0 – 3,473,943 bp on chromosome 9B in KNE 796-S that originated from 9A, and the region 60,400,000 bp – chromosome end on 6B that originated from 6A were included in the A-genome analysis. Similarly, the regions 46,980,000 – chromosome end on 6A and 0 – 3,110,118 bp on 9A were included in the B-genome analysis. The overall repeat content of the B genome was 121.6 Mb higher than that of the A genome, 119.0 Mb of which corresponded to LTR-RTs (Supplementary Table 3). LTR-RTs thus fully account for the difference in size between the A and B genomes.

A total of 182 LTR-RT families contained full-length elements. Of those, 73% comprised less than five full-length copies. Twenty-seven LTR-RT families with a minimum of five full-length copies had full-length elements in only a single subgenome (Supplementary Table 4). A total of 896 full-length elements belonging to 19 families and representing a total of 7.5 Mb were specific to the B genome compared to only 78 elements from eight families representing a total length of 0.7 Mb that were A-genome specific (Supplementary Table 4). In addition, some families had full-length elements in both subgenomes but element distribution was biased towards one subgenome. The total number of full-length elements present across these families was, however, similar in the A and B genomes (1463 *versus* 1481) (Supplementary Table 5).

Based on the number of variants present between the two LTRs of full-length elements, the insertion dates of the TEs were calculated^4^. The average age of the LTR-RT families ranged from 0 – 4.8 million years (MY). When considering only the 12 families that contained at least 80 full-length LTR-RT elements, the average age varied from ~100,000 years to 2.46 MY (Supplementary Fig. 12). Family 12 showed the most recent burst of activity with some 70% of elements having identical LTRs. The oldest peak of activity (defined as the 1^st^ – 3^rd^ quartile values), around 1.96 – 2.70 MYA, was seen in Family 11. Of the nine families for which full-length elements were found in both the A and B genomes, six contained very young elements (Supplementary Fig. 12). In contrast, none of the three families that had full length elements in only a single subgenome showed recent activity. The average value of the first quartile of the insertion dates of these three families was 1.31 MYA and their peak of activity was around 2 MYA (Supplementary Fig. 12). We used 1.31 MYA as the approximate time of the tetraploidization event.

## **Supplementary Note 4.** **Independent mutations in a MYC-bHLH transcription factor are associated with loss of purple coloration of stigma and anthers in wild and cultivated finger millet germplasm**

QTL analysis of stigma/anther color as a binomial trait with white stigma/yellow anthers being scored as 1 and purple stigma/anthers as 2 in 122 MD-20 x Okhale-1 F_2_ progeny identified a large-effect QTL (LOD = 43.9) on chromosome 4A that explained 77.0% of the variation and spanned ~1.6 Mb (5,672,174 Mb – 7,298,569 Mb). Of the 187 high confidence (HC) and 39 low confidence (LC) genes that were annotated within the ~1.6 Mb QTL region, only ELECO.r07.4AG0307750 had a description or gene ontology (GO)-term associated with anthocyanin biosynthesis. Comparative analyses showed that the start codon of ELECO.r07.4AG0307750 (4A: 6,551,367 – 6,555,024 Mb), a MYC-bHLH transcription factor orthologous to the maize anthocyanin regulatory gene *R1*, was annotated incorrectly in the KNE 796-S genome assembly because the gene lacked exon 1 and part of intron 1 in the KNE 796-S accession used for generation of the genome assembly. Analysis of *E. indica* assembly HZ-2018^3^ showed that a full-length gene copy was present on scaffold187 (Genbank Acc. Number QEPD01000187; position 139,789-144,867). Alignment of the Illumina resequencing reads of MD-20 and Okhale-1 to the full-length *E. indica* ortholog of ELECO.r07.4AG0307750 showed that Okhale-1 carried a full-length gene copy while MD-20 carried a 3’ truncated gene copy which lacked most of exon 7, intron 7, exon 8 and an additional 332 bp downstream of the TGA stop codon (deletion corresponding to the region 6,554,229 – 6,555,356 Mb in the KNE 796-S assembly). This was confirmed by amplicon sequencing. The 4B homoeolog, ELECO.r07.4BG0338780, was also misannotated in the KNE 796-S v1.0 release with the true start codon being located 966 bp upstream of the bioinformatically annotated start codon. The misannotation was caused by the presence of a stop codon in exon 1 (position 6,465,126) and a single base (G) deletion in exon 2 in KNE 796-S (position 6,466,328). Both resequencing data and amplicon sequencing showed that the two function-inactivating mutations were also present in Okhale-1. MD-20, on the other hand, carried the wild-type SNPs at those positions, but carried a 1-bp insertion (G) 35-bp downstream of the 1-bp deletion that characterized KNE 796-S and Okhale-1 (position 6,466,363). MD-20, which has white stigma and yellow anthers, therefore has non-functional alleles for the single copy MYC-bHLH transcription factor at both the 4A and 4B loci, while Okhale-1 has a single functional allele at the 4A locus, which we will refer to as the *PP* gene.

**
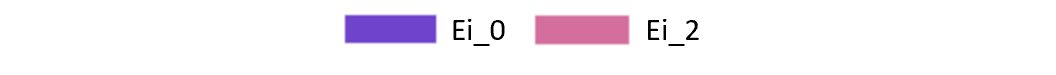
**

**Supplementary Figure 1. Coverage analysis of the alignment of *E. indica* reads (samples Ei_0 and Ei_2) to the 18 finger millet chromosomes.** The coverage analysis5 identifies the A-genome chromosomes through the presence of uniformly high coverage while the B-genome chromosomes have consistently low coverage except for repetitive regions. It also identifies the presence of reciprocal translocations between homoeologous chromosomes 6A and 6B, and 9A and 9B, as seen through the abrupt shifts in coverage between the A and B chromosomes (indicated with orange triangles). Normalized coverage computed in 16 kbp intervals using indexcov v0.2.15. Source data are available from <https://doi.org/10.6084/m9.figshare.22762430>^6^.


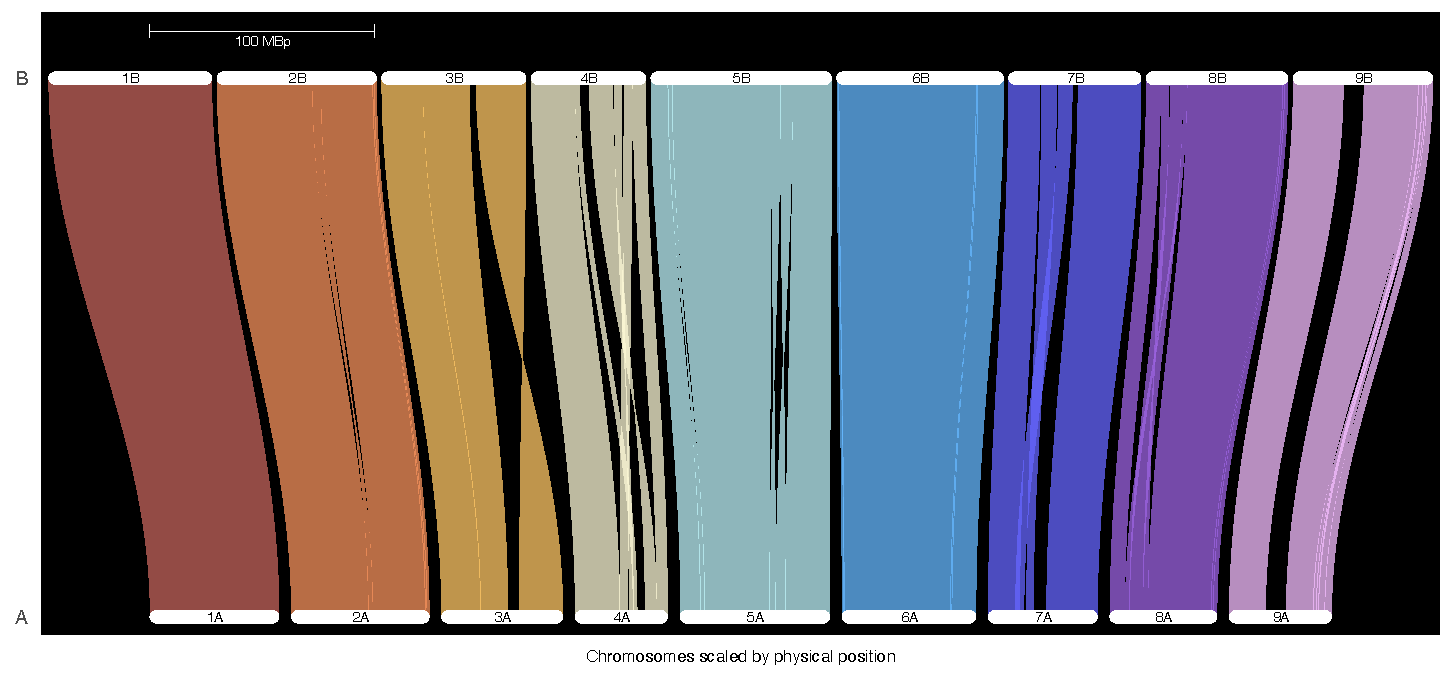


**Supplementary Figure 2. Comparative relationship between the A- and B-genome chromosomes of finger millet.** White horizontal bars represent the nine A genome (bottom) and nine B genome (top) chromosomes. Homoeologous genes are connected by lines, which each color representing a different homoeologous chromosome group. Source data are provided as a Source Data file.

| 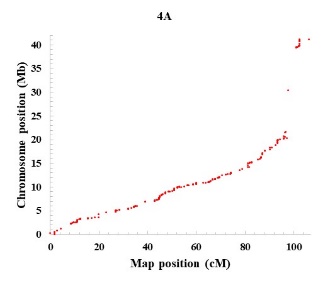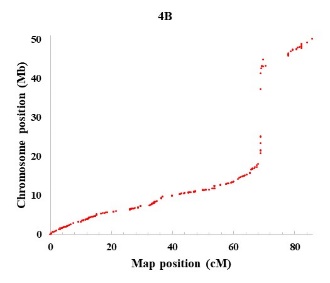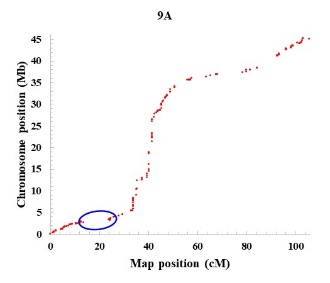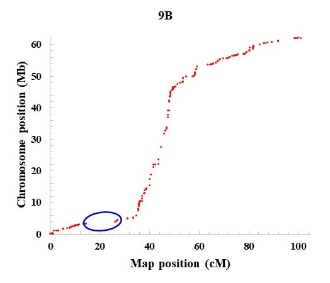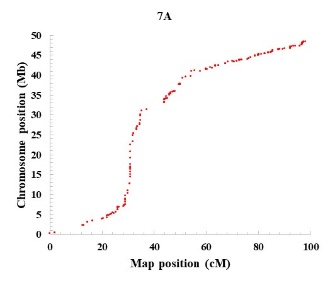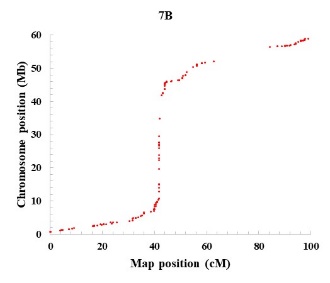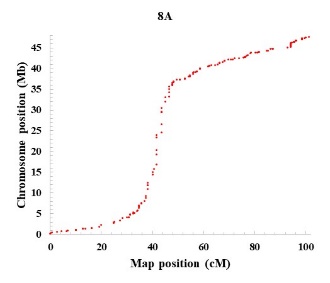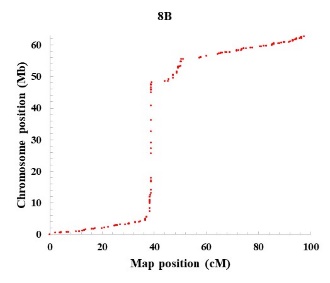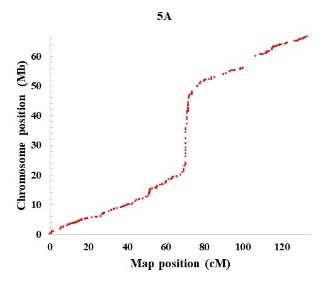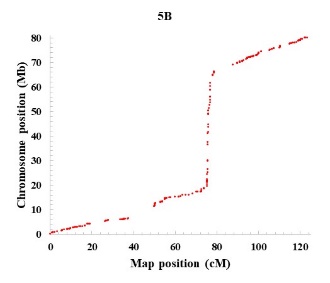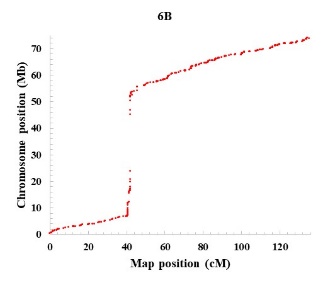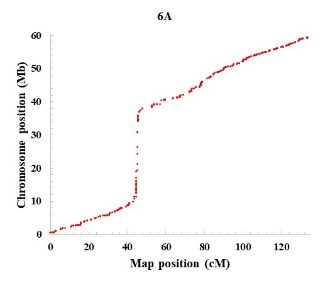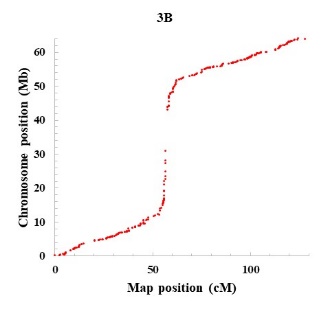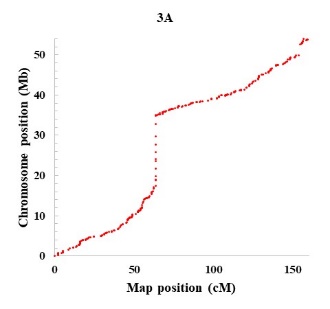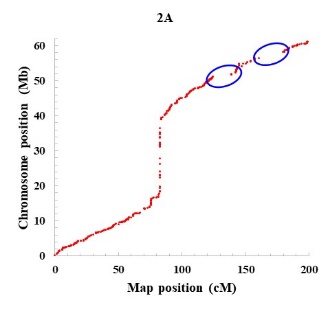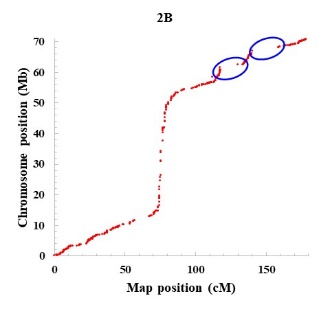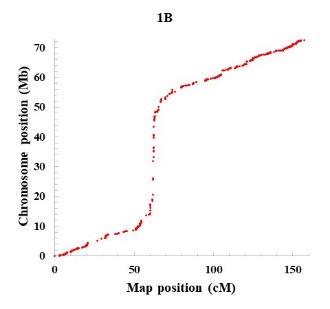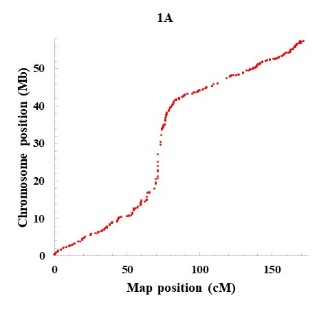 |
| --- |
|  |
| **Supplementary Figure 3.** **Correlation between genetic and physical distance.** Comparison of the location of markers (represented by red dots) on the genetic map generated in the MD-20 x Okhale-1 population (X-axis) and in the genome assembly of *Eleusine coracana* acc. KNE 796-S v1.0 (Y-axis). Regions with compressed recombination indicate the likely positions of the centromeres. Blue ovals indicate regions of apparent high recombination that correspond to breakpoints of the interstitial 2A/2B translocation and the terminal 9A/9B translocation that were present in only one of the two mapping parents. Source data are provided as a Source Data file. |


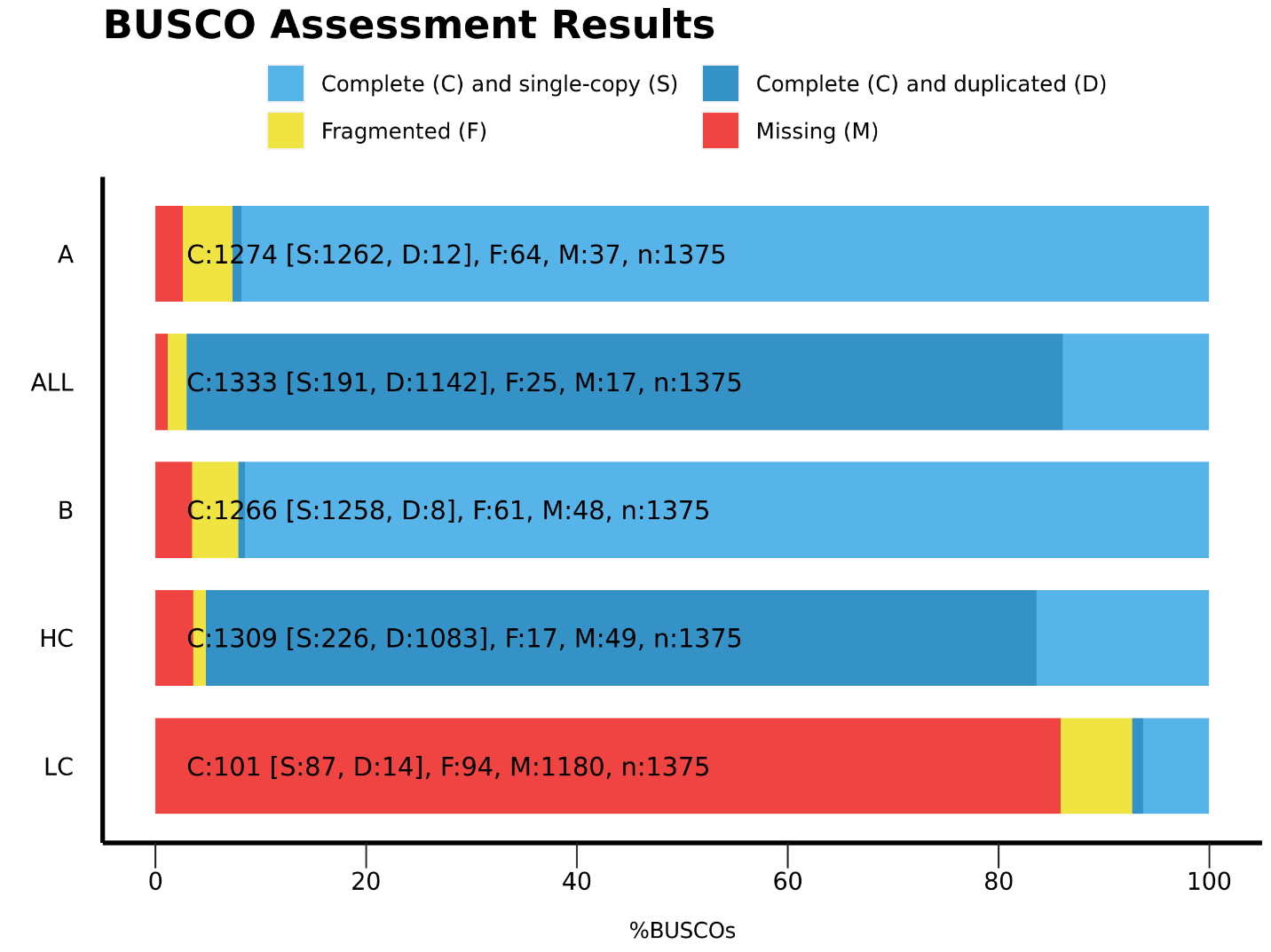


## **Supplementary Figure 4. BUSCO assessment results**

**Supplementary Figure 5. Results of population structure analyses across wild and cultivated lines.** Graphs showing the percentage contribution of finger millet accessions to the wild subpopulation (blue) and cultivated subpopulation (orange) based on population structure analysis with (A) 6185 A-genome SNPs and (B) 4592 B-genome SNPs. The B-genome analysis was conducted on 308 accessions that had been morphologically identified as E. coracana subsp. coracana and 22 accessions identified as E. coracana subsp. africana. The A-genome analysis was conducted on the same 330 accessions plus five E. indica accessions. Both analyses largely separated wild subsp. africana (and E. indica) accessions from cultivated subsp. coracana germplasm. Source data are provided as a Source Data file.

**Supplementary Figure 6. Results of population structure analyses of cultivated lines.** Graphs showing the percentage contribution of cultivated finger millet accessions (accessions with ≤75% contribution to the wild subpopulation in Supplementary Figure 5) to **(A)** two subpopulations comprising largely African (blue) and Asian (orange) accessions when populations structure was conducted with 6185 A-genome SNPs and **(B)** four subpopulations comprising largely Ethiopian (blue), a mix of Indian and African (orange), and other African lines (grey and yellow) when population structure was conducted with 4592 B-genome SNPs. Source data are provided as a Source Data file.

******Supplementary Figure 7. Principal Coordinates Analyses (PCoA).** Principal Coordinates Analysis (PCoA) showing 206 wild and cultivated finger millet accessions color-coded based on **(A)** their subpopulation membership as determined by a STRUCTURE analysis using 3000 A genome SNPs and 3000 B genome SNPs selected from the total SNP set to maximize diversity and **(B)** the presence/absence of the 9A/9B translocation. In (A), red corresponds to pop0-pop0 (wild), green to pop1-pop1 (Ethiopian), dark blue to pop1-pop2 (mixed African 1), yellow to pop1-pop3 (mixed African 2), magenta to pop1-pop4 (mixed African 3) and light blue to pop2-pop2 (Asian). In (B), green represents absence of the 9A/9B translocation, blue represents presence of the translocation, and grey indicates accessions that have not been tested. Only individuals that have >75% membership to a single subpopulation are shown. The first and second eigenvectors of the PCoA explained 22% and 9% of the genetic variability, respectively. Source data are provided as a Source Data file.

## **
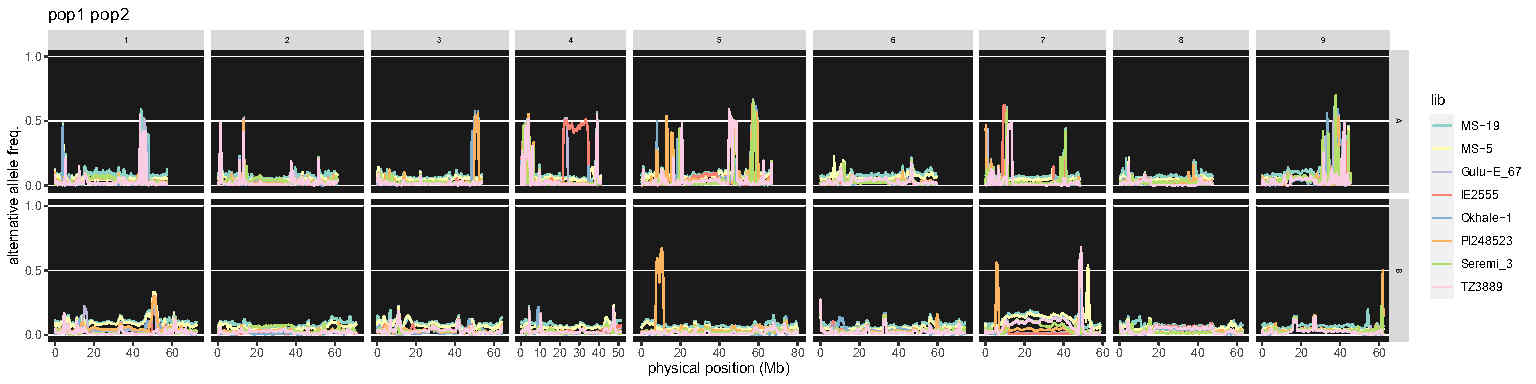

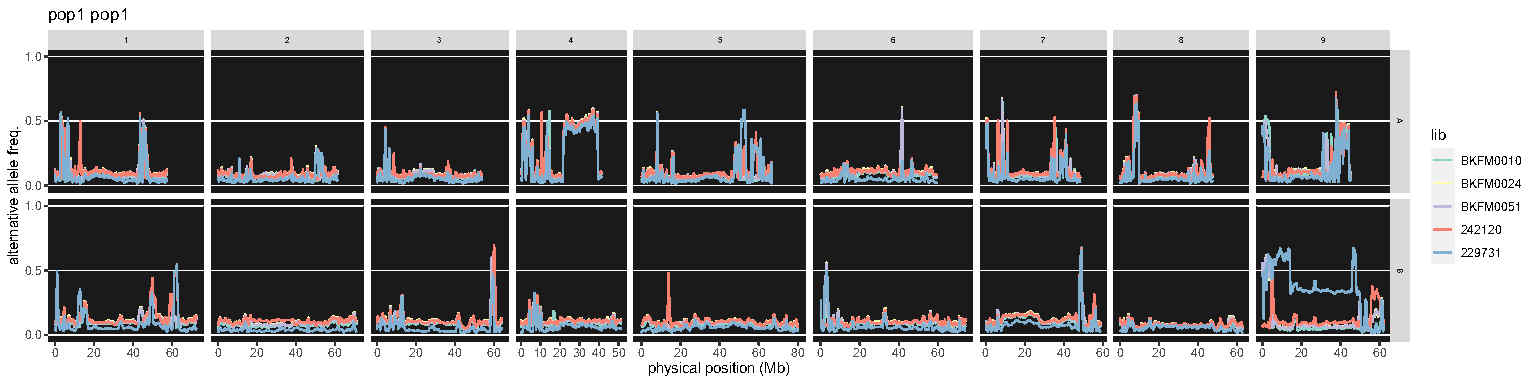

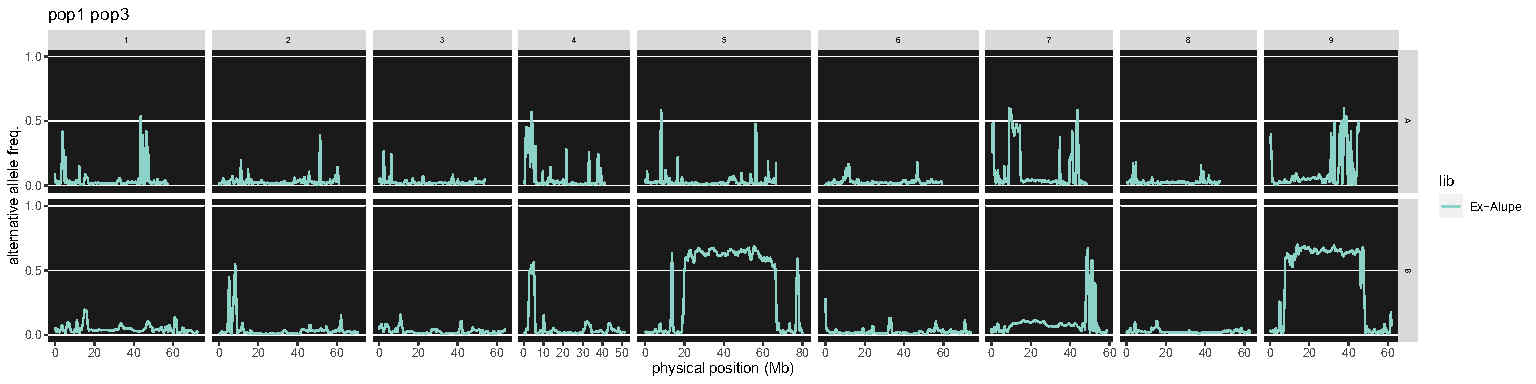

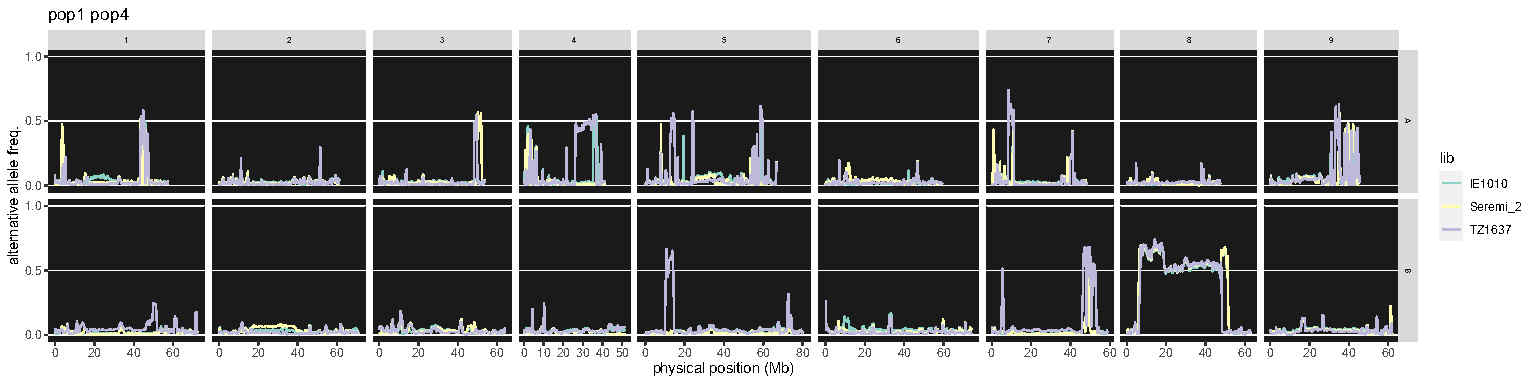

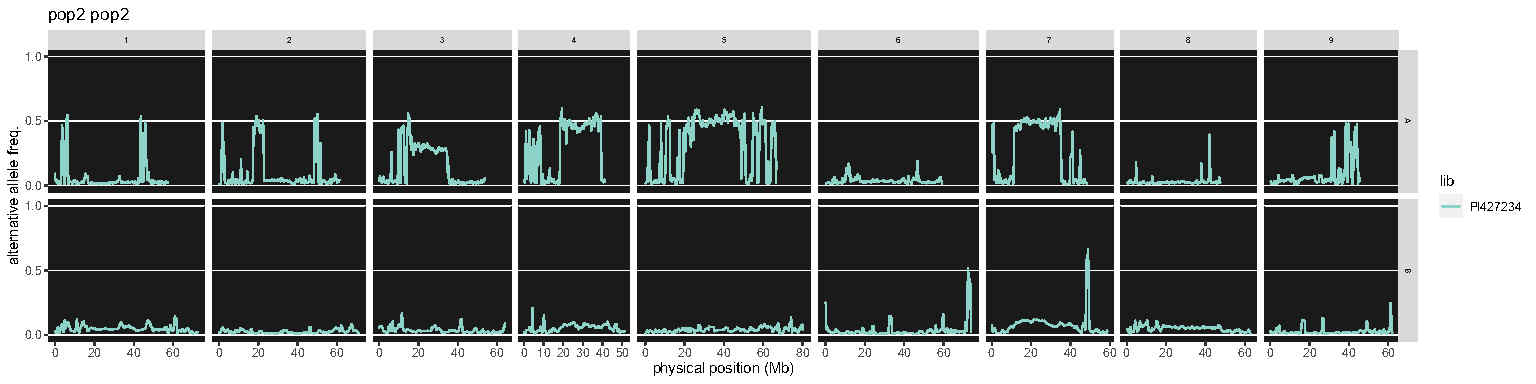
Supplementary Figure 8. Plots showing the alternate allele frequencies (Y-axis) along each chromosome (X-axis) in resequenced cultivated finger millet accessions organized by subpopulation.** Source data are provided as a Source Data file.


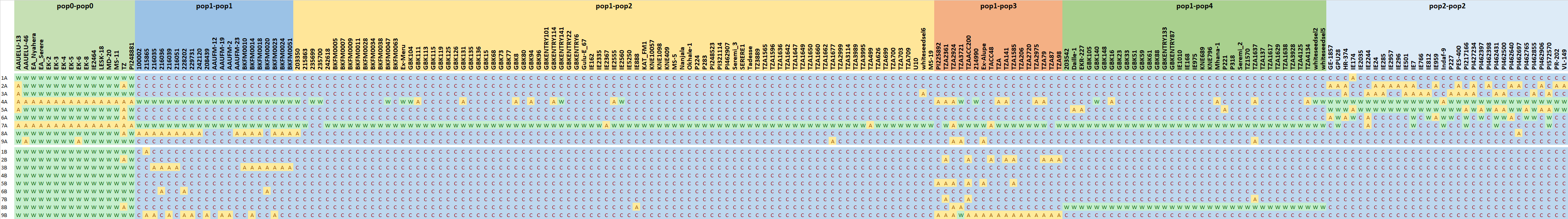


**Supplementary Figure 9. Results of population structure analyses by chromosome.** Schematic representation of the membership (>75%) of wild, Asian and African lines belonging to one of the six population groups identified on the basis of all SNPs (Figure 2) to either a ‘wild’ subpopulation (W; green) or ‘cultivated’ population (C; blue) at k=2 when conducting STRUCTURE analyses on a chromosome-by-chromosome basis. Each row represents the results of a single chromosome. Lines with ≤75% contribution to either population are labeled as admixed (A; yellow). For chromosomes 4A and 7A, which are admixed in the wild pop0-pop0 population, wild alleles were assigned as described in the main text and summarized in Supplementary Table 8. Source data are provided as a Source Data file.


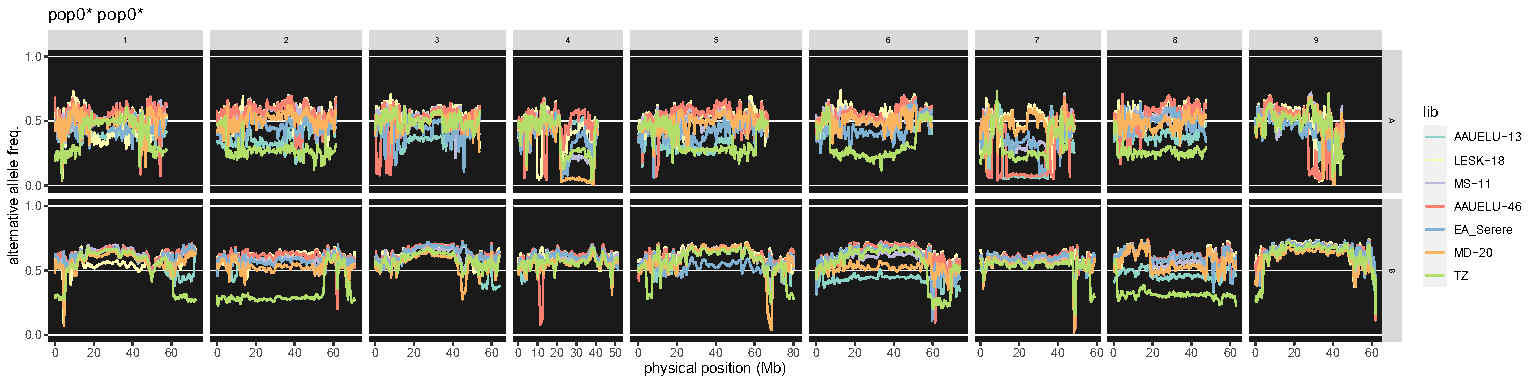

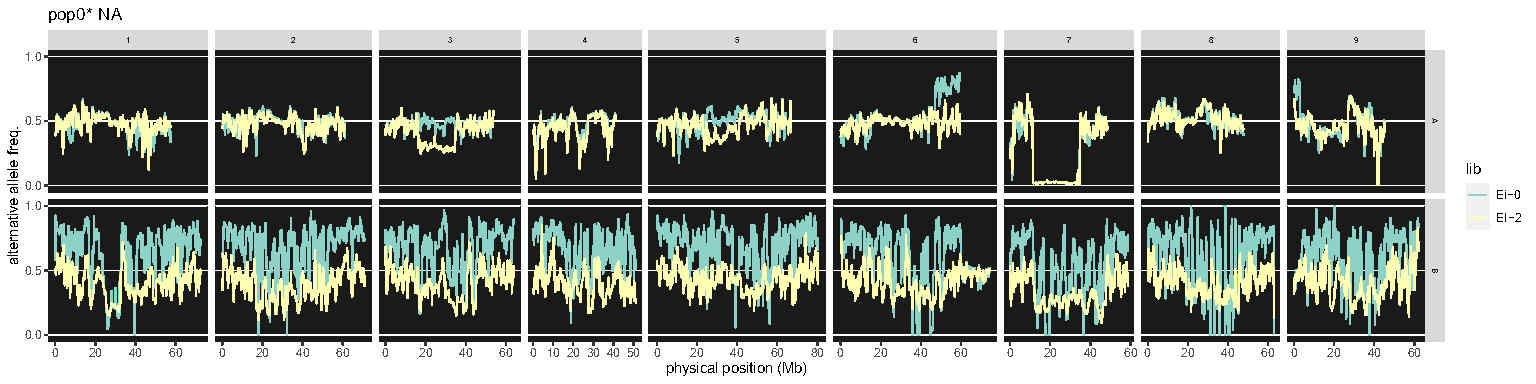


**Supplementary Figure 10. Plots showing the alternate allele frequencies (Y-axis) along each chromosome (X-axis) in resequenced wild finger millet accessions (pop0*pop0) and *E. indica* accessions (pop0*NA).** The B-genome pattern in the *E. indica* lines Ei­­_0 and Ei_2 (diploid AA) is caused by the occasional alignment of A-genome reads to the B-genome. Source data are provided as a Source Data file.


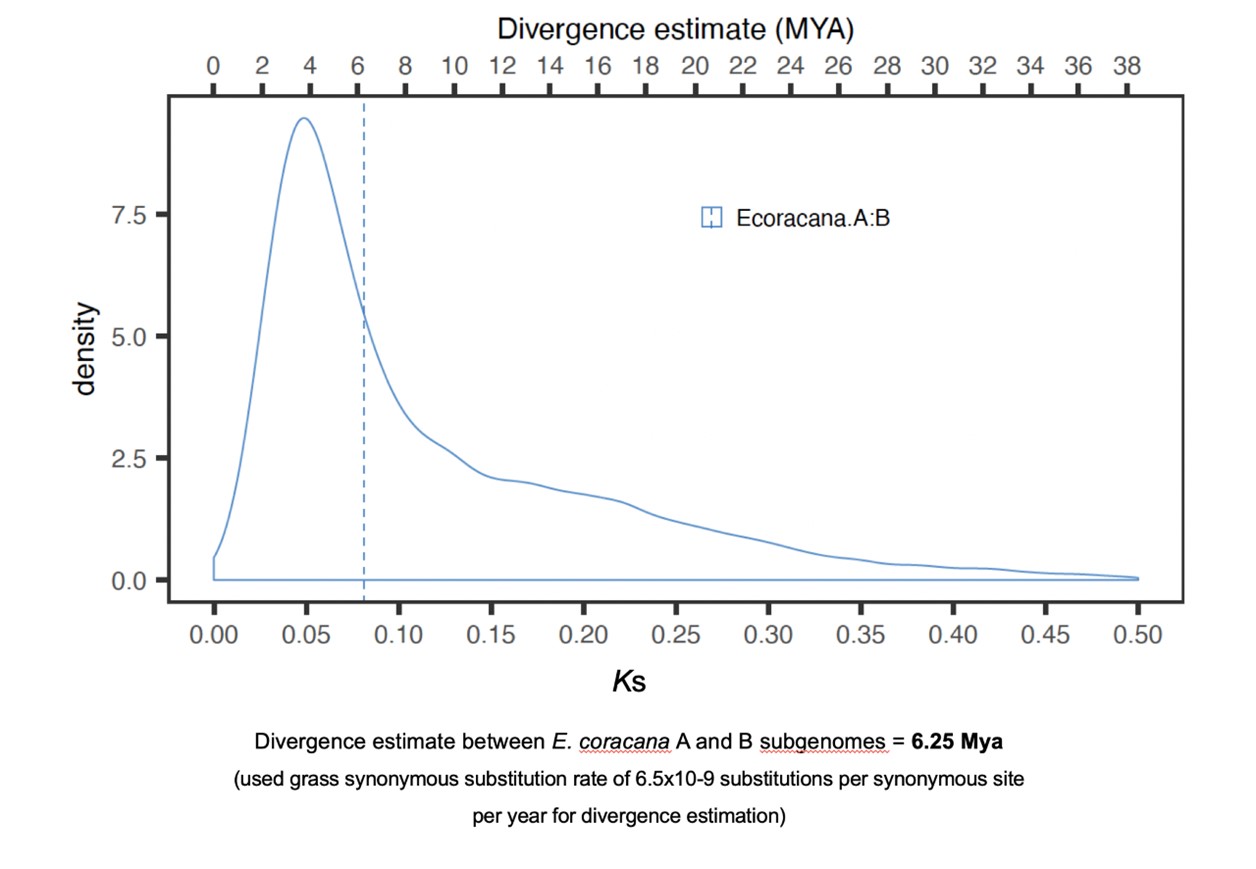


**Supplementary Figure 11. Divergence estimate between the finger millet A and B subgenomes.** Source data are provided as a Source Data file.

**Supplementary Figure 12. LTR-retrotransposon family insertion dates.** Box Plots showing the insertion dates (Y-axis; in million years) across 12 LTR-RT families (X-axis) with >80 full-length elements (Fam1: n=1585; Fam2: n=395; Fam3: n=372; Fam4: n=318; Fam5: n=282; Fam6: n= 208; Fam7: n=181; Fam8: n=139; Fam9: n=118; Fam10: n=113; Fam11: n=95; Fam12: n=92). Families 2, 6 and 10 are B-genome specific. The other families are present in both the A and B genomes. Colored boxes represent the data between the first and third quartiles with X denoting the mean and the horizontal line dividing the box the median. The whiskers represent 1.5x interquartile range, with outliers (dots). Source data are provided as a Source Data file.

**
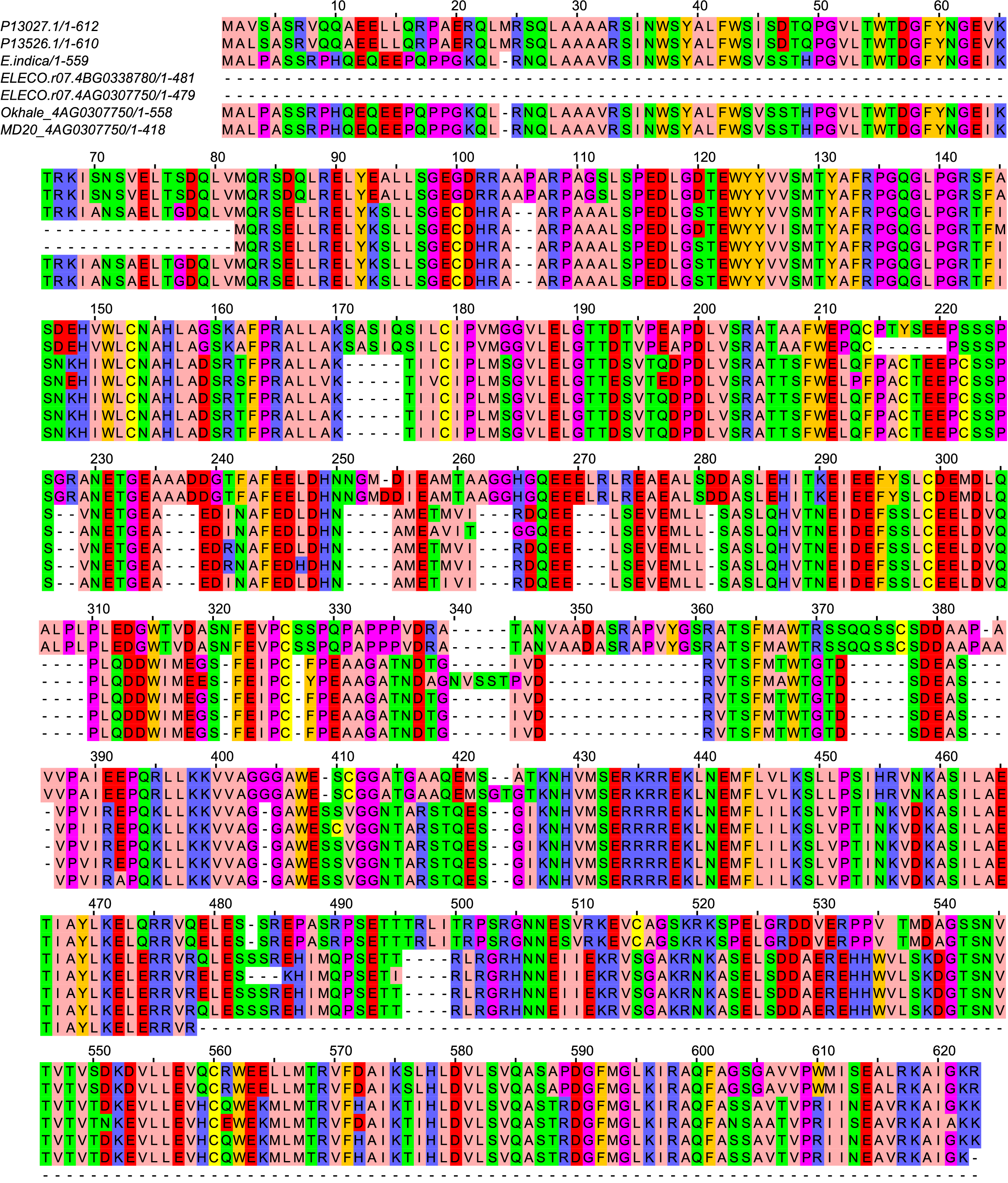
**

**Supplementary Figure 13. Multisequence alignment (MSA) at the protein level of maize *R*-S orthologs from select grass species, including finger millet.** MSA using Jalview^7^ of maize *R-*S (P13027) and maize *Lc* (P13526), and of the proteins encoded by ELECO.r07.4AG0307750 (from KNE 796-S v1.0), its 4B homoeolog ELECO.r07.4BG0338780 *(*from KNE 796-S v1.0), the *E. indica* ortholog (extracted from *E. indica* assembly HZ-2018 (Genbank Acc. Number QEPD01000187)^3^, and the corresponding 4A alleles from accessions Okhale-1 (Okhale_4AG0307750) and MD-20 (MD20_4AG0307750).


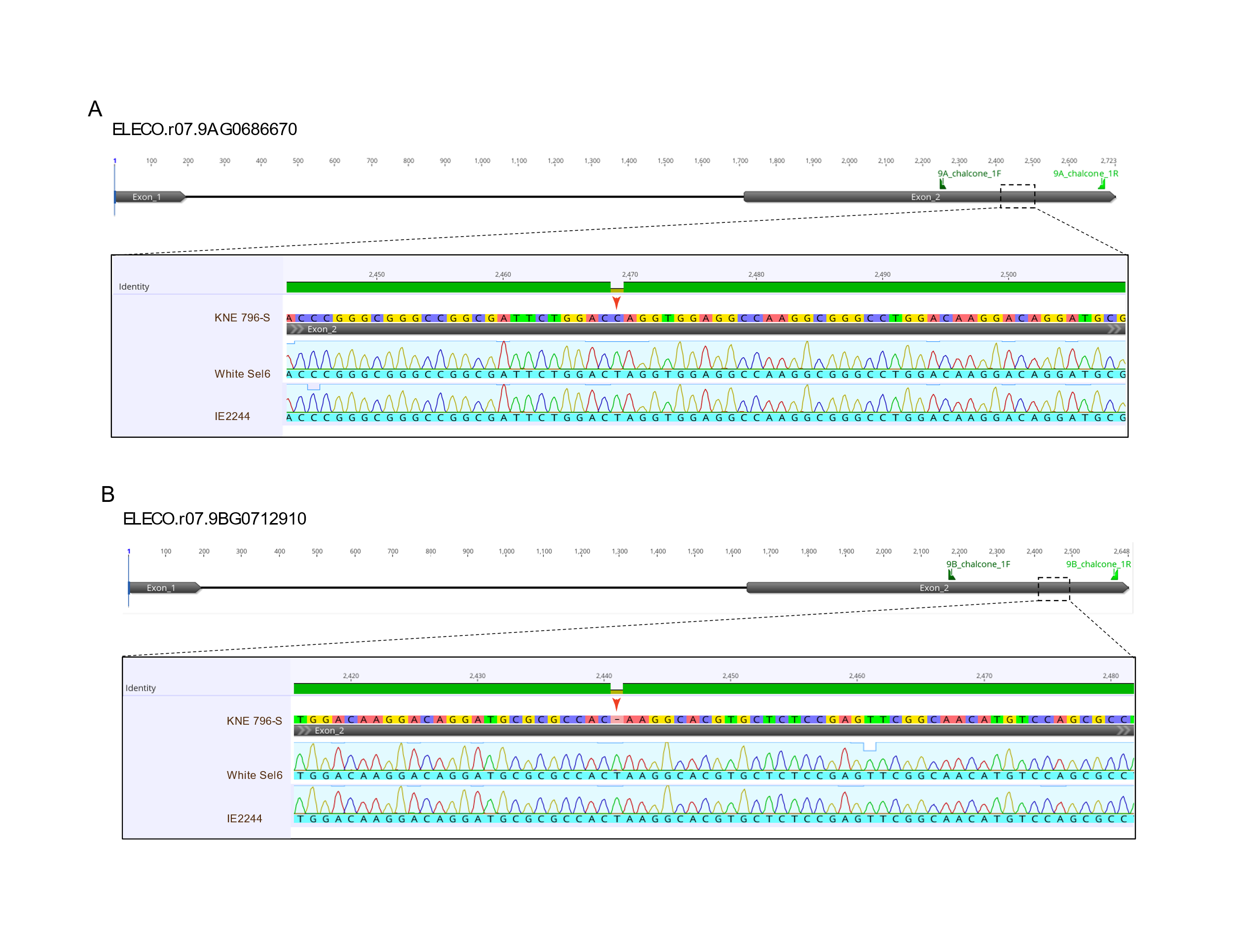


**Supplementary Figure 14. Mutations in the chalcone synthase gene in finger millet.** Chromatogram showing the presence of function-inactivating mutations (indicated with red arrowhead) in the homoeologous chalcone synthase genes ELECO.r07.9AG0686670 (A) and ELECO.r07.9BG0712910 (B) in accessions White Sel6 and IE2244 relative to the KNE 796-S reference genome.

**
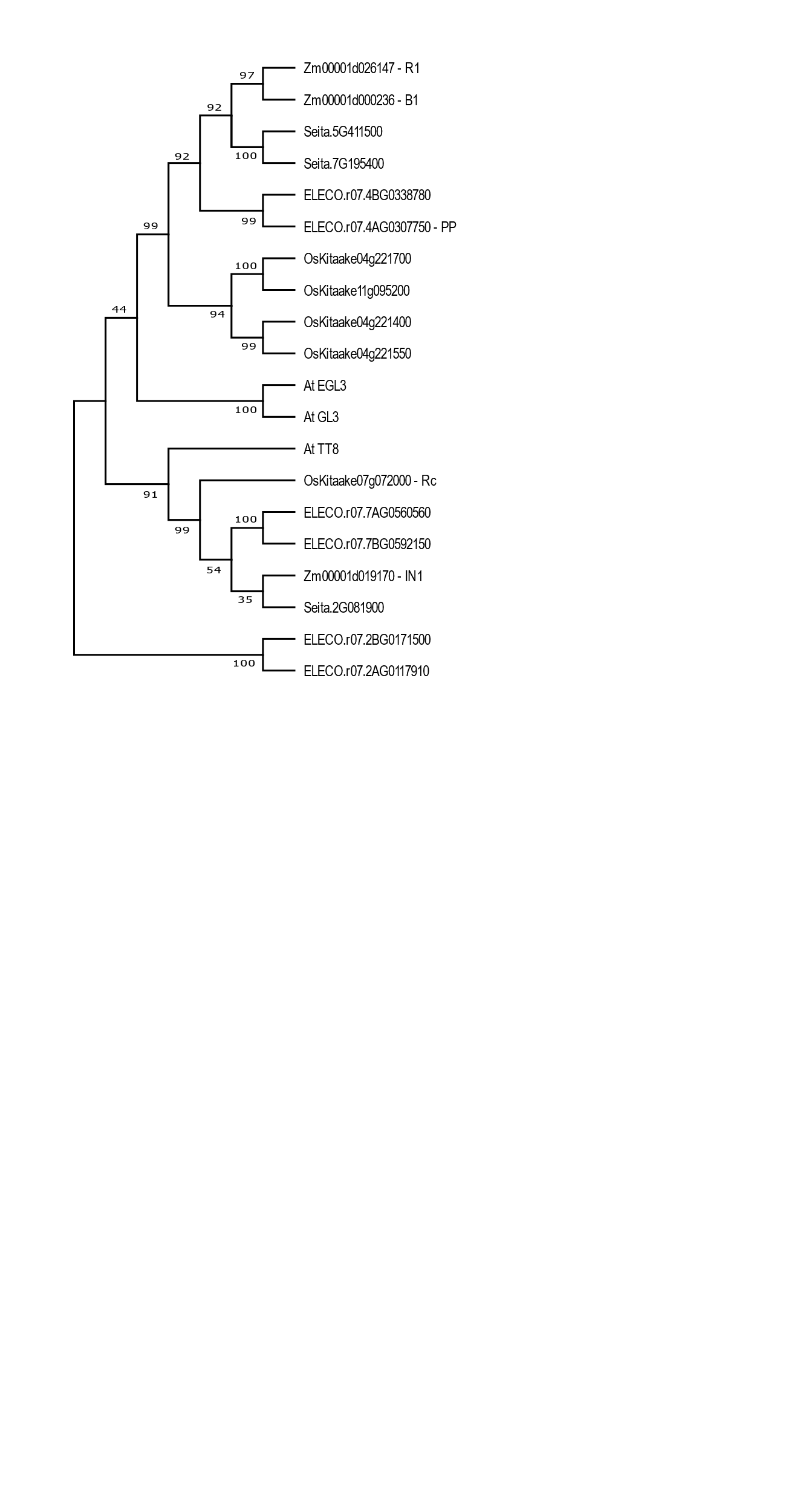
**

**Supplementary Figure 15.** **Phylogenetic relationship of bHLH proteins.** Maximum likelihood tree showing the phylogenetic relationship between bHLH family members GL3, EGL3 and TT8 from *Arabidopsis* (At), and their orthologs in finger millet (ELECO), foxtail millet (Seita), rice (OsKitaake) and maize (Zm). Support for the topology of the tree is provided by the bootstrap values at each node. Source data are provided as a Source Data file.

## **Supplementary Table 1. Contig N50 length for select publicly available Chloridoid genome assemblies.**

| **Species** | **Contig N50 in kb** |
| --- | --- |
| *Oropetium thomaeum* | 2400 |
| *Eragrostis tef* | 1550 |
| *Eleusine coracana* ML-365^8^ | 24 |
| *Eleusine coracana* PR202^9^ | 285 |
| *Eleusine coracana* KNE 796-S | 15,300 |

## **Supplementary Table 2. Final summary assembly statistics for the KNE 796-S chromosome-scale assembly.**

| Scaffold total | 532 |
| --- | --- |
| Contig total | 674 |
| Scaffold sequence total | 1111.7 Mb |
| Chromosome Sequence | 1078.8 Mb |
| Contig sequence total | 1110.3 Mb (0.1% gap) |
| Scaffold N/L50 | 9/61.3 Mb |
| Contig N/L50 | 25/15.3 Mb |

## **Supplementary Table 3. Prevalence of Gypsy and Copia LTR-RTs across the A and B subgenomes.**

| **Super_family** | **A_masked_length (bp)** | **B_masked_length (bp)** |
| --- | --- | --- |
| Gypsy | 134,208,007 | 207,987,810 |
| Copia | 61,157,819 | 90,424,486 |
| Non-specified LTR-RTs | 31,361,683 | 47,287,486 |
| LINEs and SINEs | 13,890,602 | 13,480,881 |
| DNA elements | 27,386,677 | 30,444,988 |
| Total | 268,004.788 | 389,625,651 |

## **Supplementary Table 4. Subgenome-specific LTR-RT families.**

| **Subgenome** | **LTR-RT Family ID** | **Number of intact copies in A** | **Number of intact copies in B** | **Total number of intact copies in assembly** |
| --- | --- | --- | --- | --- |
| A | 24 | 18 | 0 | 18 |
|  | 25 | 17 | 0 | 17 |
|  | 29 | 12 | 0 | 12 |
|  | 30 | 10 | 0 | 10 |
|  | 41 | 6 | 0 | 6 |
|  | 45 | 5 | 0 | 5 |
|  | 49 | 5 | 0 | 5 |
|  | 50 | 5 | 0 | 5 |
| B | 2 | 0 | 395 | 395 |
|  | 6 | 0 | 208 | 208 |
|  | 10 | 0 | 113 | 113 |
|  | 19 | 0 | 26 | 26 |
|  | 21 | 0 | 21 | 21 |
|  | 23 | 0 | 20 | 20 |
|  | 26 | 0 | 16 | 16 |
|  | 27 | 0 | 13 | 13 |
|  | 28 | 0 | 13 | 13 |
|  | 31 | 0 | 10 | 10 |
|  | 32 | 0 | 9 | 9 |
|  | 33 | 0 | 8 | 8 |
|  | 34 | 0 | 8 | 8 |
|  | 37 | 0 | 7 | 7 |
|  | 38 | 0 | 7 | 7 |
|  | 43 | 0 | 6 | 6 |
|  | 44 | 0 | 6 | 6 |
|  | 46 | 0 | 5 | 5 |
|  | 47 | 0 | 5 | 5 |

## **Supplementary Table 5.** **Intact elements from 11 LTR-RT families present in both subgenomes but displaying a subgenome bias towards insertion/amplification.**

| **Number of intact copies in A** | **Number of intact copies in B** | **Chi^2^1^** | **Family ID** | **Total copy number in assembly** | ***p*-value** | **BH^2^ adjusted *p*-value** | **Dominant subgenome** |
| --- | --- | --- | --- | --- | --- | --- | --- |
| 893 | 692 | 12.66872 | 1 | 1585 | 0.000372 | 0.000775 | A |
| 263 | 109 | 32.4438 | 3 | 372 | 1.23E-08 | 4.38E-08 | A |
| 123 | 195 | 7.804424 | 4 | 318 | 0.005212 | 0.007239 | B |
| 49 | 132 | 18.68568 | 7 | 181 | 1.54E-05 | 4.82E-05 | B |
| 76 | 42 | 4.43095 | 9 | 118 | 0.035293 | 0.038362 | A |
| 5 | 87 | 43.40262 | 11 | 92 | 4.46E-11 | 2.23E-10 | B |
| 1 | 76 | 44.50033 | 13 | 77 | 2.54E-11 | 1.59E-10 | B |
| 2 | 66 | 36.30444 | 14 | 68 | 1.69E-09 | 7.03E-09 | B |
| 10 | 59 | 17.65039 | 15 | 69 | 2.65E-05 | 6.64E-05 | B |
| 38 | 2 | 18.11912 | 17 | 40 | 2.08E-05 | 5.76E-05 | A |
| 3 | 21 | 6.206061 | 20 | 24 | 0.012731 | 0.016752 | B |

^1^ Two-way chisquare test

^2^ Benjamini-Hochberg (BH) adjusted *p*-value

## **Supplementary Table 6.** **Detailed statistics of the finger millet genome gene annotation.**

|  | **All Genes** | **HC^1^ Genes** | **LC^2^ Genes** |
| --- | --- | --- | --- |
| Number of genes | 73,012 | 48,836 | 24,176 |
| Number of mono-exonic genes | 23,263 | 11,937 | 11,326 |
| Summed cDNA lengths (bp) | 79,924,270 | 61,335,795 | 18,588,475 |
| Average cDNA length (bp) | 1,094.67 | 1,255.95 | 768.88 |
| Summed CDS lengths (bp) | 79,924,270 | 61,335,795 | 18,588,475 |
| Average CDS length (bp) | 1,094.67 | 1,255.95 | 768.88 |
| Number of mono-exonic transcripts | 23,263 | 11,937 | 11,326 |
| Average length of mono-exonic transcripts (bp) | 770.69 | 999.34 | 529.69 |
| Total number of exons | 314,217 | 246,542 | 67,675 |
| Average number of exons per transcript | 4.3 | 5.05 | 2.8 |
| Average exon length | 254.36 | 248.78 | 274.67 |
| Average Intron length | 390.71 | 366.87 | 499.09 |
| Average number of CDS exons per transcript | 4.3 | 5.05 | 2.8 |
| Summed CDS exon lengths (bp) | 79,924,270 | 61335795 | 18,588,475 |
| Average CDS exon length (bp) | 254.36 | 248.78 | 274.67 |
| Summed CDS intron lengths (bp) | 94,000,375 | 72333935 | 21,666,440 |
| Average CDS intron length (bp) | 389.71 | 365.87 | 498.09 |
| Average intergenic distance | 12,828.65 | 19,953.47 | 44,258.56 |
|  |  |  |  |
| Number [Percentage] complete BUSCOs | 1333 [97.0%] | 1309 [95.2%] | 101 [7.3%] |
| Number [Percentage] of complete/single-copy BUSCOs | 191 [13.9%] | 226 [16.4%] | 87 [6.3%] |
| Number [Percentage] of complete/duplicated BUSCOs | 1142 [83.1%] | 1083 [78.8%] | 14 [1.0%] |
| Number [Percentage] of fragmented BUSCOs | 25 [1.8%] | 17 [1.2%] | 94 [6.8%] |
| Number [Percentage] of missing BUSCOs | 17 [1.2%] | 49 [3.6%] | 1375 [85.9%] |
|  |  |  |  |
| Number of complete proteins | 72,859 | 48,836 | 24,023 |
| Number of proteins with only START codon | 48 | 0 | 48 |
| Number of proteins with only STOP codon | 48 | 0 | 48 |
| Average protein length | 365 | 418.66 | 256.41 |

^1^ HC = High confidence

^2^ LC = Low confidence

## **Supplementary Table 7. Percentage of genes present on both subgenomes or a single subgenome.**

| **Assembly** | **BUSCO** | **Percentage of genes present on both subgenomes/single subgenome** |
| --- | --- | --- |
| ML-365^8^ | ND^1^ | ND^1^ |
| PR202^9^ | 96.5% | 56.3%/43.6% |
| KNE 796-S | 97% | 83.1%/13.9% |

^1^ ND=No data available

## **Supplementary Table 8.** **Presence of pericentromeric wild segments in cultivated subpopulations.**

| **Chromosomal location of pericentromeric wild segments** | **Subpopulation(s)** |
| --- | --- |
| 4A | pop1-pop1, pop2-pop2 |
| 5A | pop2-pop2 |
| 7A | pop1-pop1, pop1-pop2, pop1-pop3, pop1-pop4 |
| 8B | pop1-pop4 |
| 9B | pop1-pop3 |

## **Supplementary Table 9. Divergence between finger millet subpopulations.**

| **Pop0-Pop0** | **Pop1-Pop1** | **Pop1-Pop2** | **Pop1-Pop3** | **Pop1-Pop4** | **Pop2-Pop2** |  |
| --- | --- | --- | --- | --- | --- | --- |
| 0.000 | 0.827 | 0.828 | 0.789 | 0.823 | 0.796 | **Pop0-Pop0** |
| 0.666 | 0.000 | 0.070 | 0.137 | 0.077 | 0.141 | **Pop1-Pop1** |
| 0.629 | 0.176 | 0.000 | 0.127 | 0.050 | 0.156 | **Pop1-Pop2** |
| 0.564 | 0.285 | 0.168 | 0.000 | 0.128 | 0.217 | **Pop1-Pop3** |
| 0.604 | 0.281 | 0.094 | 0.251 | 0.000 | 0.156 | **Pop1-Pop4** |
| 0.644 | 0.282 | 0.184 | 0.277 | 0.276 | 0.000 | **Pop2-Pop2** |

Pairwise population Dxy values are above the diagonal and Fst values are below the diagonal

**Supplementary Table 10****.** **Chromosome fusions during Chloridoideae and Panicoideae evolution from a grass ancestor with n = 12.** Ancestral chromosome numbering follows that of rice.

| Chromosome reduction from n=12 to: | Chloridoideae | Panicoideae^1^ |
| --- | --- | --- |
| *n* = 10 | 6 – 9 – 6 | 7 – 9 – 7 |
| *n* = 10 | 2 – 10 – 2 | 3 – 10 – 3 |
|  |  |  |
| Chromosome reduction from n=10 to: | *Eleusine* | *Paniceae*^1^ |
| *n* = 9 | 5 – 12 – 5 | 12 – 5 – 12 |

^1^ From Devos^10^ and Daverdin *et al.*^11^

## **Supplementary Table 11.** **Two-way MANOVA test criteria and results for subgenome effect on transcript levels in 12,030 homoeologous gene pairs in 10 tissue-specific transcriptome datasets.**

## **Supplementary Table 12. Stigma color, genotypic score at the *PP* locus and seed catechin level in MD-20, Okhale-1 and 14 F_2_ progeny from the cross MD-20 x Okhale-1.**

| **Accession ID** | **Genotype at locus ELECO.r07.4AG0307750^1^** | **Stigma color** | **Catechin level (g/kg)^2^** |
| --- | --- | --- | --- |
| MD-20 | B | White | 2.71 ± 0.07 |
| Okhale-1 | A | Purple | 1.62 ± 0.03 |
| F2-19 | A | Purple | 1.16 ± 0.04 |
| F2-35 | B | White | 1.22 ± 0.02 |
| F2-9 | B | White | 1.24 ± 0.01 |
| F2-28 | A | Purple | 1.25 ± 0.08 |
| F2-137 | A | Purple | 1.26 ± 0.06 |
| F2-54 | A | Purple | 1.53 ± 0.06 |
| F2-12 | B | White | 1.58 ± 0.04 |
| F2-52 | A | Purple | 1.94 ± 0.02 |
| F2-104 | B | White | 2.07 ± 0.15 |
| F2-58 | A | Purple | 2.30 ± 0.02 |
| F2-149 | B | White | 2.40 ± 0.06 |
| F2-42 | A | Purple | 2.84 ± 0.03 |
| F2-82 | B | White | 3.35 ± 0.05 |
| F2-77 | A | Purple | 4.36 ± 0.02 |

^1^ Genotypic information obtained from mapping data (**Supplementary Data 13)**

^2^ Average (n=3) catechin levels in seed and standard deviation

## **Supplementary Table 13. Genome assemblies used in comparative analyses with finger millet and their download sources.**

| **Genome assembly** | **Grass subfamily** | **Source** |
| --- | --- | --- |
| *Oryza sativa* v7.0 | Ehrhartoideae | Phytozome (<https://phytozome-next.jgi.doe.gov>) |
| *Oropetium thomaeum* v1.0 | Chloridoideae | Phytozome (<https://phytozome-next.jgi.doe.gov>) |
| *Eragrostis tef* v3 | Chloridoideae | CoGe (<https://genomevolution.org/CoGe>) |
| *Sorghum bicolor* v3.1.1 | Panicoideae | Phytozome (<https://phytozome-next.jgi.doe.gov>) |

## **Supplementary Table 14. Primer sequences used to amplify specific genes or gene regions.**

| **Gene – Target region** | **Primer ID** | **Location in genome sequence** | **Sequence (5’ to 3’)** | **Annealing temp. (^0^C)** | **Extension time (sec.)** |
| --- | --- | --- | --- | --- | --- |
| ELECO.r07.4AG0307750 - Upstream deletion | 4A307740F_UpstrFul^1^ | Not in KNE 796-S genome | GTCCCGACCTCATCAGGAAC | 55 | 30 |
|  | 4A307750_UpstrDel^2^ | 4A: 6,550,029-6,550,049 | CCTGAAGAATCTGATCGGTAG | 55 | 30 |
|  | 4A307750R_Upstr | 4A: 6,550,419-6,550,398 | TGACCAGTAGTTTGATCTAACG |  |  |
| ELECO.r07.4AG0307750 – 3’ MD-20 deletion | MD_20_Del_F_2.2^3^ | 4A: 6,554,010-6,554,028 | GAGAAGGCGACGGGAGAAG | 60 | 90 |
|  | MD_20_Del_R_2.2 | 4A: 6,555,444-6,555,421 | TCAATTCATAGACCATCCAAACAG |  |  |
| ELECO.r07.4AG0307750 – Entire gene^4^ | PP_4A_F1 | 4A: 6,549,986-6,550,005 | CGGGGTTTGTTCCAATTCAG | 58 | 75 |
|  | PP_4A_R1.2 | 4A: 6,550,562-6,550,542 | CTTTCATCATCACCAGGTACC |  |  |
|  | PP_4A_F2 | 4A: 6,550,385-6,550,410 | ATGAAATCTATACCGTTAGATCAAAC | 50 | 90 |
|  | PP_4A_R2 | 4A: 6,551,483-6,551,465 | CTCGGTGCTCCCCAGATC |  |  |
|  | PP_4A_F3 | 4A: 6,551,241-6,551,265 | CCTCTCTATAGTTAATGATCGGAAG | 58 | 75 |
|  | PP_4A_R3 | 4A: 6,552,431-6,552,408 | GGATTTCGATTTTAGAATTGCTGC |  |  |
|  | PP_4A_F4 | 4A: 6,552,272-6,552,295 | AATGCATACTTACTTGGAAATTGC | 58 | 75 |
|  | PP_4A_R4 | 4A: 6,553,310-6,553,284 | GCAGATATCTTAAACTAGATTGCTATC |  |  |
|  | PP_4A_F5 | 4A: 6,553,093-6,553,112 | CATCGAAGTGGTTGTTGGAG | 58 | 75 |
|  | PP_4A_R5 | 4A: 6,554,236-6,554,254 | TGTGTTCCCTGCTGCTCG |  |  |
|  | PP_4A_F6 | 4A: 6,554,075-6,554,101 | ACAAGGTAATAATAATGCAAAAGTTAC | 50 | 90 |
|  | PP_4A_R6 | 4A: 6,555,443-6,555,421 | CAATTCATAGACCATCCAAACAG |  |  |
| ELECO.r07.4BG0338780 - indel verification^5^ | 4B338780F | 4B: 6,465,992-6,466,011 | AGAACAGCGAGCAGTTACGG | 60 | 30 |
|  | 4B338780R | 4B: 6,466,464-6,466,446 | GTACCACTCGGTGTCGCCG |  |  |
| ELECO.r07.4BG0338780 – SNP verification^6^ | 4B338780StopF | 4B: 6,465,052-6,465,076 | ATTTTCATAACATCTTCACTTTCTG | 50 | 30 |
|  | 4B338780StopR | 4B: 6,465,340-6,465,319 | TCTTTTCCCATCGATGTCGTAC |  |  |
| 9A/9B translocation | 9AT_F | 9A: 3,127,492-3,127,509 | CCTGCGCTCTCTTCGCCG | 65 | 30 |
|  | 9BT_F | 9B: 3,765,283-3,765,301 | CCCTGCGCTCTCTTCGCCA | 65 | 30 |
|  | 9A_R2F^7^ | 9A: 3,127,641-3,127,621 | GCTACCTACTTCGAATCTTCG |  |  |
| ELECO.r07.9AG0686670 – SNP verification^8^ | 9A_chalcone_1F | 9A: 35,420,171-35,420,187 | CAGCTCGTCTCCGCGTC | 65 | 30 |
|  | 9A_chalcone_1R | 9A: 35,420,618-35,420,602 | CGCTGTGGAGCACGACA | 65 | 30 |
| ELECO.r07.9BG0712910 – 1bp insertion verification | 9B_chalcone_1F | 9B: 53,692,386-53,692,402 | CAGCTCGTCTCCGCGGG | 65 | 30 |
|  | 9B_chalcone_1R | 9B: 53,692,833-53,692,817 | CGCTGTGGAGCACGACG | 65 | 30 |

^1^ Designed in Exon 1 of ELECO.r07.4AG030775 (*PP* gene) in *E. indica* acc. HZ-2018^3^ (Genbank Acc. QEPD01000187). This primer site is absent from the KNE 796-S reference genome due to a deletion that encompasses the primer site. 4A307740F_UpstrFull in combination with 4A307750R_Upstr amplifies a 511 bp fragment if the 5’ region of ELECO.r07.4AG030775 is intact.

^2^ 4A307750_UpstrDel in combination with 4A307750R_Upstr amplifies a 391 bp fragment from the lines that carry a 5’ deletion in the *PP* gene.

^3^ MD_20_Del_F_2.2/ MD_20_Del_R_2.2 amplifies a 1435 bp fragment from the genotypes with an intact *PP* gene and a 308 bp fragment from genotypes carrying the 1127 bp 3’ deletion identified in MD-20.

^4^ Overlapping sets of primers were designed to amplify and sequence the entire gene in select cultivars. Each primer pair amplifies an approximately 1100 bp fragment and amplicons overlap by ~150 bp on each side.

^5^ 4B338780F/4B338780R amplifies a 473 bp fragment in cultivated accessions and a 475 bp fragment in the wild accessions. This primer set was used to validate the two 1-bp indels in exon 2 in ELECO.r07.4BG0338780 that are differentially present in the wild and cultivated accessions analyzed.

^6^ 4B338780StopF/4B338780StopR amplifies a 289 bp fragment and was used to validate a SNP leading to a stop codon in exon 1 that was present in the cultivated lines tested and absent in the wild lines tested.

^7^ Primer 9A_R2F is located on chromosome 9A in the KNE 796-S reference sequence in a region that originates from chromosome 9B (KNE 796-S carries a 9A/9B translocation). 9AT_F/9A_R2F will therefore amplify if the 9A/9B translocation is present. 9BT_F/9A_R2F will amplify if the 9A/9B translocation is absent.

^8^ The primers in the chalcone synthase genes were used to validate the mutations identified in IE2244 and White Sel6 presented in Supplementary Fig. 14.

**Supplementary references**

1 Qi, P. *et al.* UGbS-Flex, a novel bioinformatics pipeline for imputation-free SNP discovery in polyploids without a reference genome: Finger millet as a case study. *BMC Plant Biol* **18**, 117 (2018).

2 Pendergast, T. H., Qi, P., Odeny, D. A., Dida, M. M. & Devos, K. M. A high-density linkage map of finger millet provides QTL for blast resistance and other agronomic traits. *The Plant Genome* **15**, e20175 (2022).

3 Zhang, H. *et al.* Development of a goosegrass (*Eleusine indica*) draft genome and application to weed science research. *Pest Manag Sci* **75**, 2776-2784 (2019).

4 SanMiguel, P., Gaut, B. S., Tikhonov, A., Nakajima, Y. & Bennetzen, J. L. The paleontology of intergene retrotransposons of maize : dating the strata. *Nature Genet.* **20**, 43-45 (1998).

5 Pedersen, B. S., Collins, R. L., Talkowski, M. E. & Quinlan, A. R. Indexcov: fast coverage quality control for whole-genome sequencing. *GigaScience* **6**, 1-6 (2017).

6 Schatz, M. Supporting data for "Genome analyses reveal population structure and a purple stigma color candidate gene in finger millet' by Devos *et al*. 2023. *Figshare*  [https://doi.org/10.6084/m9.figshare.22762430](https://urldefense.com/v3/__https:/doi.org/10.6084/m9.figshare.22762430__;!!NLFGqXoFfo8MMQ!qDsNyjzzpnNPx2N0yviggNRvq5V47DF4DVMblMXkZHg4JGeeO9Hjd1vXSm8qa8OHWLHVtAAPqn6X-EQ7HQ$) (2023).

7 Waterhouse, A. M., Procter, J. B., Martin, D. M., Clamp, M. & Barton, G. J. Jalview Version 2--a multiple sequence alignment editor and analysis workbench. *Bioinformatics* **25**, 1189-1191 (2009).

8 Hittalmani, S. *et al.* Genome and transcriptome sequence of finger millet (*Eleusine coracana* (L.) Gaertn.) provides insights into drought tolerance and nutraceutical properties. *BMC Genomics* **18**, 465 (2017).

9 Hatakeyama, M. *et al.* Multiple hybrid *de novo* genome assembly of finger millet, an orphan allotetraploid crop. *DNA Research* **25**, 39-47 (2017).

10 Devos, K. M. Updating the 'Crop Circle'. *Current Opinion in Plant Biology* **8**, 155-162 (2005).

11 Daverdin, G. *et al.* Comparative relationships and chromosome evolution in switchgrass (*Panicum virgatum*) and its genomic model, foxtail millet (*Setaria italica*). *Bioenergy Research* **8**, 137-151 (2015).
