# Supplementary material for: Genome analyses reveal population structure and a purple stigma color gene candidate in finger millet
Source: Nat Commun. 2023 Jun 21;14:3694. doi: 10.1038/s41467-023-38915-6 (PMC10284860; doi:10.1038/s41467-023-38915-6)
Supplement: Supplementary file 3 — Description of Additional Supplementary Files [file 41467_2023_38915_MOESM3_ESM.pdf]

Description of Additional Supplementary Files

File Name: Supplementary Data 1
Description: A-genome SNPs used in population structure and PCoA analyses

File Name: Supplementary Data 2
Description: B-genome SNPs used in population structure and PCoA analyses

File Name: Supplementary Data 3
Description: Summary information on accessions

File Name: Supplementary Data 4
Description: Allele frequencies in 1000 SNP windows with 0=AA, 0.5=AB and 1=BB for 27 resequenced finger millet accessions belonging to the six subpopulations identified on the basis of GBS-SNPs

File Name: Supplementary Data 5
Description: Fst and Dxy values between subpopulations on a chromosome-by-chromosome basis for the A genome

File Name: Supplementary Data 6
Description: Fst and Dxy values between subpopulations on a chromosome-by-chromosome basis for the B genome

File Name: Supplementary Data 7
Description: Fst values between subpopulations on a chromosome-by-chromosome basis for the A and B genomes

File Name: Supplementary Data 8
Description: Dxy values between subpopulations on a chromosome-by-chromosome basis for the A and B genomes

File Name: Supplementary Data 9
Description: Finger millet homoeologous gene pairs with a 1:1 relationship

File Name: Supplementary Data 10
Description: Unique single copy genes present in the finger millet A genome

File Name: Supplementary Data 11
Description: Unique single copy genes present in the finger millet B genome

File Name: Supplementary Data 12
Description: Gene pairs and their transcript levels in different tissues used for the assessment of subgenome dominance

File Name: Supplementary Data 13
Description: Genotypic and phenotypic data used for genetic mapping and QTL analysis
