# Supplementary material for: Genome analyses reveal population structure and a purple stigma color gene candidate in finger millet
Source: Nat Commun. 2023 Jun 21;14:3694. doi: 10.1038/s41467-023-38915-6 (PMC10284860; doi:10.1038/s41467-023-38915-6)
Supplement: Supplementary file 17 — Reporting Summary [file 41467_2023_38915_MOESM17_ESM.pdf]

Corresponding author(s): Katrien M. Devos

Last updated by author(s): May 18, 2023

Reporting Summary

Nature Portfolio wishes to improve the reproducibility of the work that we publish. This form provides structure for consistency and transparency in reporting. For further information on Nature Portfolio policies, see our Editorial Policies and the Editorial Policy Checklist.

Statistics

For all statistical analyses, confirm that the following items are present in the figure legend, table legend, main text, or Methods section.

n/a	Confirmed	
☐	☒	The exact sample size (*n*) for each experimental group/condition, given as a discrete number and unit of measurement
☒	☐	A statement on whether measurements were taken from distinct samples or whether the same sample was measured repeatedly
☐	☒	The statistical test(s) used AND whether they are one- or two-sided<br>*Only common tests should be described solely by name; describe more complex techniques in the Methods section.*
☐	☒	A description of all covariates tested
☐	☒	A description of any assumptions or corrections, such as tests of normality and adjustment for multiple comparisons
☐	☒	A full description of the statistical parameters including central tendency (e.g. means) or other basic estimates (e.g. regression coefficient) AND variation (e.g. standard deviation) or associated estimates of uncertainty (e.g. confidence intervals)
☐	☒	For null hypothesis testing, the test statistic (e.g. *F*, *t*, *r*) with confidence intervals, effect sizes, degrees of freedom and *P* value noted<br>*Give P values as exact values whenever suitable.*
☐	☒	For Bayesian analysis, information on the choice of priors and Markov chain Monte Carlo settings
☐	☒	For hierarchical and complex designs, identification of the appropriate level for tests and full reporting of outcomes
☒	☐	Estimates of effect sizes (e.g. Cohen's *d*, Pearson's *r*), indicating how they were calculated

Our web collection on statistics for biologists contains articles on many of the points above.

Software and code

Policy information about availability of computer code

Data collection	No software was used for data collection.
Data analysis	The following software packages were used for data analysis: MECAT v1.4, QUIVER v2.1, JUICER v1.8.9, BWA-mem v0.7.17-r1188, GATK v3.6-0-g89b7209, GMATA v2.3, LTR-finder v1.1, LTRharvest v1.6.2, LTR_retriever v2.9.0, SINE-scan v1.1, MGEscan-nonLTR v2.0, MITE-Hunter v1.0, MITE Tracker v1.0, HelitronScanner v1.0, RepeatMasker v4.0.7, GenomeThreader v1.7.1, HISAT2 v2.0.4, StringTie v1.2.3, Cuffcompare v2.2.1, Transdecoder v3.0.0, Augustus v3.3.2, EVidenceModeller v1.1.1, BUSCO v3.02, AHRD (no version number listed), ClustalO v1.2.2, PAL2NAL v14, PAML v4.10.3, Trim Galore v0.45, HISAT2 v2.1.0, StringTie v2.1.1, SAS v9.4, GENESPACE v0.9.3, MCScanX (no version number listed), Bowtie2 v2.4.1, GATK v3.4, snpEff v4.3, STRUCTURE v2.3.4, GenAlEx v6.501, DnaSP6 v6.12.03, Core Hunter v2.0, R/QTL v1.52, Leica Application Suite X software v3.0.12.21488, Jalview v2.11.1.5, MEGA v11.0.13.

For manuscripts utilizing custom algorithms or software that are central to the research but not yet described in published literature, software must be made available to editors and reviewers. We strongly encourage code deposition in a community repository (e.g. GitHub). See the Nature Portfolio guidelines for submitting code & software for further information.

Data

Policy information about availability of data

All manuscripts must include a data availability statement. This statement should provide the following information, where applicable:

- Accession codes, unique identifiers, or web links for publicly available datasets
- A description of any restrictions on data availability
- For clinical datasets or third party data, please ensure that the statement adheres to our policy

The datasets generated during this study are available from NCBI's Sequence Read Archive (PACBIO, Illumina sequencing reads and annotated genome assembly for KNE 796-S: BioProject PRJNA838475; Illumina sequencing reads for other finger millet accessions: BioProject PRJNA838475; Ion Proton sequencing reads: BioProject PRJNA876392; GBS reads: BioProject PRJNA870151). The annotated KNE 796 S genome assembly is also available from Phytozome (https://phytozome-next.jgi.doe.gov/info/Ecoracana_v1_1). The following datasets retrieved from NCBI's SRA were used as part of the study: PRJNA377606 (RNAseq), PRJNA648385 (RNAseq) and SRP136342 (GBS data of MD-20 x Okhale-1 mapping population). Seed of the sequenced finger millet accession, KNE 796-S, has been deposited in the U.S. National Plant Germplasm System (NPGS) under accession number PI 702583. Distribution of the other finger millet germplasm may be restricted due to country-of-origin specific regulations and seed stock limitations. Please contact K.M. Devos (kdevos@uga.edu) for further information. Seed from non-restricted germplasm will be distributed, pending availability and unforeseen circumstances, within 3 weeks of receiving the request. All other datasets have been submitted as Supplementary Information. Source data are provided with this paper or are available from https://doi.org/10.6084/m9.figshare.22762430 (Ref 105).

Research involving human participants, their data, or biological material

Policy information about studies with human participants or human data. See also policy information about sex, gender (identity/presentation), and sexual orientation and race, ethnicity and racism.

Reporting on sex and gender	N/A
Reporting on race, ethnicity, or other socially relevant groupings	N/A
Population characteristics	N/A
Recruitment	N/A
Ethics oversight	N/A

Note that full information on the approval of the study protocol must also be provided in the manuscript.

Field-specific reporting

Please select the one below that is the best fit for your research. If you are not sure, read the appropriate sections before making your selection.

☒ Life sciences ☐ Behavioural & social sciences ☐ Ecological, evolutionary & environmental sciences

For a reference copy of the document with all sections, see nature.com/documents/nr-reporting-summary-flat.pdf

Life sciences study design

All studies must disclose on these points even when the disclosure is negative.

Sample size	The number of progeny from the cross MD-2O x Okhale-1 analyzed was determined by seed availability. The germplasm collection analyzed was assembled to have representation from the main finger millet growing regions and with input from finger millet breeders and geneticists.
Data exclusions	As described in the manuscript, four F2 progeny were excluded because of the large number of predicted double recombination events in these lines, a common outcome of seed or DNA contamination.
Replication	Duplicated samples were included in the GBS analysis. In the rare case that a discrepancy between duplicates was observed, genotyping was repeated.
Randomization	Randomization is not applicable given that there was no replication and there were no treatments in our study.
Blinding	Investigators were blinded as to the origin of finger millet accessions during the analyses (grouping was a result of the analysis and not an a priori known).

Reporting for specific materials, systems and methods

We require information from authors about some types of materials, experimental systems and methods used in many studies. Here, indicate whether each material, system or method listed is relevant to your study. If you are not sure if a list item applies to your research, read the appropriate section before selecting a response.

Materials & experimental systems

n/a	Involved in the study	
☒	☐	Antibodies
☒	☐	Eukaryotic cell lines
☒	☐	Palaeontology and archaeology
☒	☐	Animals and other organisms
☒	☐	Clinical data
☒	☐	Dual use research of concern
☐	☒	Plants

Methods

n/a	Involved in the study	
☒	☐	ChIP-seq
☒	☐	Flow cytometry
☒	☐	MRI-based neuroimaging

Dual use research of concern

Policy information about dual use research of concern

Hazards

Could the accidental, deliberate or reckless misuse of agents or technologies generated in the work, or the application of information presented in the manuscript, pose a threat to:

No	Yes	
☒	☐	Public health
☒	☐	National security
☒	☐	Crops and/or livestock
☒	☐	Ecosystems
☒	☐	Any other significant area

Experiments of concern

Does the work involve any of these experiments of concern:

No	Yes	
☒	☐	Demonstrate how to render a vaccine ineffective
☒	☐	Confer resistance to therapeutically useful antibiotics or antiviral agents
☒	☐	Enhance the virulence of a pathogen or render a nonpathogen virulent
☒	☐	Increase transmissibility of a pathogen
☒	☐	Alter the host range of a pathogen
☒	☐	Enable evasion of diagnostic/detection modalities
☒	☐	Enable the weaponization of a biological agent or toxin
☒	☐	Any other potentially harmful combination of experiments and agents
